# Associations between omega-3 fatty acids and 25(OH)D and psychological distress among Inuit in Canada

**DOI:** 10.1080/22423982.2017.1302684

**Published:** 2017-06-17

**Authors:** Hans-Ragnar Skogli, Dominique Geoffroy, Hope A. Weiler, Grethe S. Tell, Laurence J. Kirmayer, Grace M. Egeland

**Affiliations:** ^a^ Department of Global Public Health and Primary Care, University of Bergen, Bergen, Norway; ^b^ Culture and Mental Health Research Unit, Lady Davis Institute, Jewish General Hospital, Montreal, QC, Canada; ^c^ School of Dietetics and Human Nutrition, McGill University, Ste-Anne-de-Bellevue, QC, Canada; ^d^ Norwegian Institute of Public Health, Bergen, Norway; ^e^ Division of Social and Transcultural Psychiatry, Department of Psychiatry, McGill University, Montreal, QC, Canada

**Keywords:** Aboriginal health, Kessler, K6, omega-3, vitamin D

## Abstract

**Background**: Inuit in Canada have experienced dietary changes over recent generations, but how this relates to psychological distress has not been investigated.

**Objective**: To evaluate how nutritional biomarkers are related to psychological distress.

**Design**: A total of 36 communities in northern Canada participated in the International Polar Year Inuit Health Survey (2007–2008). Of 2796 households, 1901 (68%) participated; 1699 Inuit adults gave blood samples for biomarker analysis and answered the Kessler 6-item psychological distress questionnaire (K6). Biomarkers included n-3 fatty acids and 25-hydroxyvitamin D (25(OH)D). The K6 screens for psychological distress over the last 30 days with six items scored on a 4-point scale. A total score of 13 or more indicates serious psychological distress (SPD). Logistic regression models were used to investigate any associations between SPD and biomarkers while controlling for age, gender, marital status, days spent out on the land, feeling of being alone, income and smoking.

**Results**: The 30-day SPD prevalence was 11.2%, with women below 30 years having the highest and men 50 years and more having the lowest SPD prevalence at 16.1% and 2.6%, respectively. SPD was associated with being female, younger age, not being married or with a common-law partner, spending few days out on the land, feelings of being alone, smoking and low income. Low levels of both 25(OH)D and long-chain n-3 FAs were associated with higher odds for SPD in both unadjusted and adjusted logistic regression models.

**Conclusion**: In this cross-sectional analysis, low levels of 25(OH)D and long-chain n-3 FAs were associated with higher odds ratios for SPD, which highlights the potential impact of traditional foods on mental health and wellbeing. Cultural practices are also important for mental health and it may be that the biomarkers serve as proxies for cultural activities related to food collection, sharing and consumption that increase both biomarker levels and psychological well-being.

**Abbreviations**: n-3 FAs: omega-3 fatty acids; PUFAs: polyunsaturated fatty acids; 25(OH)D: 25-hydroxyvitamin D; IPY: International Polar Year; IHS : Inuit Health Survey; RBC: red blood cell; OR: odds ratio; K6: Kessler 6-item screening scale; SPD: serious psychological distress; EPA: eicosapentaenoic acid (20:5 n-3); DHA: docosahexaenoic acid (22:6 n-3); DPA n-3: docosapentaenoic acid (22:5 n-3); n-3 LC-PUFAs: EPA (20:5 n-3) + DHA (22:6 n-3) + DPA (22:5 n-3); BMI: body mass index (kg m^–^^2^)

## Introduction

The potential relationship between diet and health for the Inuit, who reside in the arctic regions of Canada, Alaska and Greenland, became a focus of interest in the medical literature when Bang and Dyerberg reported an inverse relationship between omega-3 fatty acids and the incidence of cardiovascular disease [1]. The traditional Inuit diet includes country foods such as caribou and marine mammals and fish, the latter two being rich in omega-3 fatty acids and vitamin D. The shift from a traditional lifestyle to a modern-wage economy has brought with it changes in every dimension of life including physical activity and food habits, with greater consumption of market foods rich in energy but of poorer nutrient quality compared to traditional foods [2,3]. These changes in nutrition and cultural practices have been posited to account for an epidemiologic transition with increased risk for maladies typically associated with a Western lifestyle [4,5]. Mental health has also suffered during this transition as evidenced by one of the highest suicide rates in the world [6].

The traditional Inuit concept of the person has an *ecocentric* aspect, which implies human beings are in constant interaction with the environment, including the land and animals (e.g. through the collection and consuption of traditional country foods), which are thought to affect well-being [7]. Hence, diet may have biological, psychological and social implications in Inuit communities.

To better understand the potential links between diet and mental health, we investigated the demographic, social, and nutrient biomarker correlates of serious psychological distress (SPD) among Inuit in a cross-sectional health survey in the Canadian Arctic. Omega-3 fatty acids (n-3 FAs) and 25-hydroxyvitamin D (25(OH)D) were examined because of emerging evidence of their importance in mental health [8,9] and the high content of these nutrients in traditional food [10–12]. We, therefore, hypothesised that low levels of n-3 FAs and 25(OH)D would be associated with psychological distress.

## Methods

### Study population

The International Polar Year (IPY) Inuit Health Survey (IHS) was conducted in 2007 and 2008 from August through October in all 36 communities of three Inuit jurisdictions in northern Canada (Nunavut, Nunatsiavut and Inuvialuit Settlement Region). The Canadian Coast Guard Ship Amundsen visited the 33 coastal communities while land research teams conducted the survey in the three non-coastal communities The communities are located from a latitude of 54°10ʹN to 76°25ʹN. Inuit adults, 18 years or older, were invited to participate through random selection of households from each community. A total of 2796 Inuit households were approached, of which 1901 (68%) participated, with a total of 2595 participants. Details of study design and biomarker analyses are available elsewhere [13].

Nurses obtained medical histories and fasting morning blood samples from the participants, and measured height, without shoes, to the nearest millimetre using a stadiometer, and weight using a Tanita instrument (Tanita TBF-300GS with goal setter, Tanita Corporation of America, Inc., Arlington Heights, IL, USA). Due to survey logistics, fasting morning blood samples were not available for all study participants. Body mass index (BMI) was calculated (kg/m^2^) and obesity was defined as BMI ≥ 30. Respondents were interviewed about household composition, traditional food harvesting and consumption, smoking and alcohol habits, and socio-economic indicators (income, education). For the mental well-being questionnaire, participants could choose either to have an interview-administered or self-administered bilingual questionnaire. All questionnaires were available in dialects of Inuktitut current in the regions included in the survey.

### Ethics

The project was conducted through a participatory research process [3,14]. Scientific research licenses were obtained from the Nunavummi Qaujisaqtulirijikkut (Nunavut Research Institute) and the Aurora Research Institute-Aurora College (Inuvik, Northwest Territories). The McGill Faculty of Medicine Institutional Review Board provided a certificate of ethical acceptability. Information needed for informed consent was provided to potential participants through a bilingual DVD and in writing in English and in the Inuktitut dialects appropriate for the regions. Written consent was obtained from all participants.

### Biochemical analyses

Fasting red blood cell (RBC) membrane fatty acids were assessed by gas-liquid chromatography (Lipid Analytical Laboratories Inc., Guelph, Canada). RBCs were available for 84.8% of the study population. Varian 3400 gas-liquid chromatograph (Palo Alto, CA, USA) was used to analyse fatty acid methyl esters, with a 60-m DB-23 capillary column (0.32-mm internal diameter). Morning fasting blood samples were drawn and processed into serum (for 25(OH)D analyses) and RBC (for analyses of n-3 FAs). Samples were frozen at −80°C in the freezer onboard the ship. Samples from land-based teams were stored at −20°C; all samples were stored within 4–8 h after sampling. For RBC fatty analysis, 200 µl of RBC were stored in 200 µl of a 1:1 solution of methanol and distilled water plus 8.4 mg BHT (Becton Dickinson, Franklin Lakes, NJ).

The RBC membrane n-3 FAs were analysed and expressed as % of total FAs. Total n-3 FAs included α-linolenic, stearidonic, eicosatrienoic, eicosatetraenoic, eicosapentaenoic, docosapentaenoic and docosahexaenoic. Concentrations were determined by Lipid Analytical Laboratories, University of Guelph Research Park, based on the methodology of Folch et al. [15]. The FAME were prepared using standard techniques [16], replacing boron trifluoride-methanol with boron trichloride-methanol to reduce the formation artefact [17]. Vitamin D status was assessed using LIAISON total 25(OH)D assay (DiaSorin, Stillwater, MN, USA) at McGill University. This laboratory participated in the DEQAS (Vitamin D External Quality Assessment scheme) programme and received a certificate of proficiency for 2009–2010 which reflects that 80% or more of the reported results fell within 30% of the All-Laboratory Trimmed Mean (ALTM) [13].

### The Kessler K6 scale

The Kessler scale (K6) is a widely used tool for screening for anxiety or mood disorders and estimating the prevalence of common psychiatric disorders in a population. The K6 aims to identify those with a high likelihood of having a mental disorder (especially depression) according to DSM-IV criteria [18,19]. The K6 was developed through a process of structured telephone, face-to-face and clinical interviewing of the general population and those reporting symptoms of severe psychological distress. It was used by the World Health Organisation as the mental health screening tool in the Mental Health Survey Initiative and has been used in Inuit Health Surveys [20,21]. The K6 has been validated in several languages, cultures and countries [22,23]. The K6 contains six questions: “During the past 30 days, about how often did you feel: (1) anxious; (2) hopeless; (3) restless or fidgety; (4) so depressed that nothing could cheer you up; (5) that everything was an effort; (6) worthless. The respondent rates each question on a four-point scale: 0 (none of the time), 1 (a little of the time), 3 (some of the time), and 4 (all of the time). The summed score indicates the presence or absence of psychological distress with a range of 0–24. Serious psychological distress (SPD) was defined as a score ≥ 13, a cut-off point that optimally equalises between false positives and false negatives in many populations [18].

### Statistical analysis

Chi-square and Student’s *t*-tests were used to evaluate the characteristics associated with SPD. Logistic regression models assessed the association between different biomarkers and SPD in unadjusted and adjusted analysis, in which individual biomarker levels were divided into tertiles with the highest level used as the reference category. Testing for trend was done by assessing significance over tertiles in the same models. Multivariable analysis was adjusted for factors known to influence mental well-being and biomarker concentrations: [6,24–26] age, gender, marital status, days spent out on the land, feeling of being alone, income and smoking. Further, fishing or hunting during the last year and obesity were evaluated. Marital status was dichotomised into married/common law or not married (single, separated, divorced, widowed). Spending time outside of the community on the land has a central role in Inuit culture; [7] therefore we categorised the number of days spent out on the land over the last 12 months in three equally sized groups, 0–5 days, 6–27 days, and 28 days and more. Feelings of being alone were dichotomised into no and any variation of yes to the question: “Are you ever alone when you would in fact prefer to be with others?” Income was dichotomised into earning less than $20,000 or more than $20,000 (CAN). Education was coded into three categories: 1 = elementary school or less; 2 = some years of secondary school; and 3 = completed secondary school or greater. However, education was not included in our models as it was highly associated with age and income. Smoking was dichotomised into smoker or non-smoker and included as a covariate because of the known association between smoking and biomarker levels [27]. Alcohol consumption was evaluated but not included in the final analysis because it did not modify the results. We stratified on age below 50 years, and 50 years and above because of known age differences in traditional food intake and because of the potential for age differences in SPD. Data analysis was performed using Stata version 14 (StataCorp, 2015, Stata Statistical Software: Release 14. College Station, TX, USA). A p ≤ 0.05 was considered statistically significant.

## Results

In all, 1,699 individuals answered the K6 for a response rate of 65.5% of the total sample. Of these, 1,649 had their blood samples analysed for n-3 FA and 25(OH)D. Among participants who had blood analyses, 234 answered five or fewer K6 questions, while 318 did not answer any. There were no significant age or gender differences between those who answered all K6 questions and those who answered just a few or none at all. The respondents included in our analyses had a mean (SD) age of 42 (15.1) years; 71% were below 50 years of age, 61% were female, 69% were smokers, 64% were married or in a common law relationship and 48.6% had an income ≥ $20,000 (CAN). Women had significantly higher average scores on all individual K6 questions compared with men, except for questions 3 (restless) and 5 (effort). The mean (SD) Kessler score (0–24) was 5.9 (4.9) for the total sample, 4.7 (3.6) in the non-SPD (0–12) and 15.7 (2.9) in the SPD (13–24) category. The crude 30-day prevalence of SPD was 11.2% and was nearly two times higher for women (13.4%) than men (7.7%) (p < 0.001). Women between 18 and 29 years of age had the highest prevalence of SPD (16.1%) while men over 50 years of age had the lowest prevalence (2.6%). However, there was a lower SPD prevalence among older men and women.

In bivariate analyses, SPD was significantly less prevalent if the participant was a non-smoker, male, over 50 years of age, had a partner (married/common law), had an income ≥ $20,000, did not feel alone, and spent time out on the land (Table 1). In addition, fishing or hunting during the last year, higher education and higher BMI showed significantly lower SPD prevalence (χ^2^ test, p < 0.01), but the associations were not significant when tested in multivariate logistic regression models.

**Table 1.** Characteristics associated with serious psychological distress (SPD): International Polar Year Inuit Health Survey (n = 1699).

	n	% SPD	p-value*
Sex			<0.001
Male	659	7.7	
Female	1.040	13.4	
Mean age (years)			<0.001
<50	1.217	13.2	
≥50	482	6.0	
Marital status			0.002
Married/common law	602	9.4	
Not married	1.091	14.5	
Days out on the land:			−0.096
0–5, mean: 1.7	460	14.4	
6–27, mean: 13.4	464	9.1	
27–365, mean: 75.8	481	6.9	
Feeling of being alone			<0.001
Yes	852	16.9	
No	820	5.1	
Smoking			<0.001
Yes	1160	13.6	
No	534	6.0	
Income			<0.001
<$20,000	776	15.6	
>$20,000	783	6.4	
BMI (kg m^–^^2^)			
<30	1.066	13.4	<0.001
≥30	605	8.10	
Education			−0.077
≤Elementary	404	12.8	
Secondary	556	14.7	
≥Secondary	711	7.6	

*P-values for chi-square test for difference or Kendall’s tau.

### Biomarkers

Biomarker levels were significantly associated with age. The mean (SD) total n-3 FA was 5.80 (3.43) with no significant difference between men and women (data not shown), but with significant differences between age categories (p < 0.001) (Table 2). The mean (SD) 25(OH)D concentration value was 58.5 (33.9) nmol/L without any significant gender difference, but with significant differences between age categories (p < 0.001) (Table 2). Low 25(OH)D (< 50 nmol/L) values were observed for 45.7% while 28.0% had values ≥ 75 nmol/L. We observed a positive correlation between age and total n-3 FA and 25(OH)D (Pearson’s r = 0.45 and 0.54, respectively, p < 0.01).

**Table 2.** Mean (SD) biomarker levels by age group for participants who answered the Kessler 6 psychological distress assessment: International Polar Year Inuit Health Survey (N = 1,649).

		Total n-3*	EPA + DHA*	N-3 LC-PUFAs*	25(OH)D nmol/L
Age (years)	n	Mean (SD)	Mean (SD)	Mean (SD)	Mean (SD)
18–49	1173	4.91(2.62)	3.31(2.03)	4.62(2.58)	48.5(28.0)
≥ 50	476	8.01(4.12)	6.14(3.40)	7.77(4.10)	84.8(33.5)
Total	1649	5.80(3.43)	4.13(2.81)	5.52(3.40)	58.9(33.9)

*Fatty acids expressed as % of total RBC membrane fatty acid content.

### N-3 fatty acids

In the non-SPD and SPD category, average total n-3 FA values were 5.9 (SD: 3.5) and 4.9 (SD: 2.8) respectively (p < 0.01). We noted an inverse relationship (Pearson’s r =−0.14, p < 0.01) between K6 score and total n-3 FA and we found significantly different (p < 0.001) K6 scores when comparing low and high n-3 LC-PUFAs levels (Table 3). In unadjusted logistic regression analyses, low and medium tertiles of total and individual n-3 FAs were significantly associated with higher ORs for SPD relative to the highest tertile reference group (Table 4). Adjusted logistic regression models showed the same tendency, but only the lowest tertile of DHA was significantly associated with SPD. In age-stratified analyses, significant n-3 FA associations and trends were observed only for those 50 years of age or older, with low levels of EPA, EPA+DHA, n-3 LC-PUFAs and total n-3 FAs significantly associated with SPD. For total n-3 FAs and n-3 LC-PUFAs, the medium tertile was also significantly associated with SPD (Table 4).

**Table 3.** Variables by tertile for n-3 LC-PUFAs and 25(OH)D and test for trend.

	N-3 LC-PUFAs*				25(OH)D			
Tertile	1	2	3	p-value**†**	1	2	3	p-value**†**
Age (years)	36.6(13.8)	38.4(13.0)	52.2(13.9)	<0.001	32.5(10.2)	41.3(13.9)	53.1(13.7)	<0.001
% female	63.0	60.6	60.1	0.225	64.3	60.0	59.8	0.045
% married/common law	58.7	64.4	69.7	<0.001	61.8	65.9	64.9	0.114
Days out on land (SD)	25.4(43.7)	30.4(47.5)	37.4(53.9)	0.000	21.4(37.4)	33.1(48.7)	37.9(56.2)	<0.001
% feeling being alone	54.0	50.1	47.3	0.055	55.2	51.1	46.3	0.009
% smoking	77.9	73.2	58.2	<0.001	82.6	69.3	57.4	<0.001
% highest income	44.0	44.9	52.0	0.077	39.2	51.2	51.7	<0.001
K6 score (SD)	6.5(5.3)	6.1(4.8)	4.9(4.5)	<0.001	6.8(5.1)	5.9(4.9)	4.9(4.6)	<0.001

*EPA+DHA+DPA n-3

^†^ Test for trend over tertiles.

**Table 4.** ORs for SPD by selected fatty acids and 25(OH)D with high values as reference, age specified and all observations.

	<50		≥50		All observations	
	Unadjusted	Adjusted*	Unadjusted	Adjusted*	Unadjusted	Adjusted*
	n = 1173	n = 901	n = 476	n = 343	n = 1649	n = 1244
	OR (95% CI)	OR (95% CI)	OR (95% CI)	OR (95% CI)	OR (95% CI)	OR (95% CI)
Total n-3 FAs						
High	1.00	1.00	1.00	1.00	1.00	1.00
Medium	1.13 (0.73–1.73)	0.97 (0.57–1.79)	4.24 (1.17–15.35)	10.15 (1.22–83.69)	1.52 (1.00–2.31)	1.00 (0.58–1.76)
Low	1.31 (0.86–1.99)	1.34 (0.81–2.45)	5.01 (1.42–17.95)	13.43 (1.64–109.82)	2.11 (1.42–3.15)	1.58 (0.92–2.68)
p-trend^†^	0.202	0.210	0.012	0.008	<0.001	0.057
N-3 LC-PUFAs						
High	1.00	1.00	1.00	1.00	1.00	1.00
Medium	1.08 (0.65–1.54)	1.02 (0.55–1.75)	4.59 (1.18–15.45)	10.11 (1.22–83.47)	1.44 (0.94–2.19)	0.95 (0.54–1.68)
Low	1.21 (0.85–1.93)	1.26 (0.84–2.55)	5.51 (1.42–17.96)	12.82 (1.60–104.30)	2.21 (1.49–3.28)	1.67 (0.98–2.84)
P-trend^†^	0.237	0.155	0.012	0.010	<0.001	0.027
EPA+DHA						
High	1.00	1.00	1.00	1.00	1.00	1.00
Medium	1.11 (0.81–1.90)	1.11 (0.61–1.88)	3.86 (1.06–14.12)	4.37 (0.87–21.92)	1.66 (1.10–2.51)	1.13 (0.65–1.97)
Low	1.23 (0.85–1.98)	1.34 (0.73–2.28)	5.55 (1.55–19.23)	6.01 (1.26–29.29)	2.06 (1.38–3.09)	1.53 (0.88–2.66)
P-trend^†^	0.237	0.366	0.006	0.022	<0.001	0.104
EPA						
High	1.00	1.00	1.00	1.00	1.00	1.00
Medium	1.05 (0.68–1.62)	1.26 (0.70–2.24)	1.79 (0.51–6.22)	1.66 (0.38–7.34)	1.62 (1.08–2.43)	1.09 (0.64–1.86)
Low	1.31 (0.87–1.98)	1.64 (0.94–2.88)	4.98 (1.65–15.08)	4.17 (1.10–15.84)	1.78 (1.20–2.65)	1.32 (0.77–2.26)
P-trend^†^	0.197	0.080	0.002	0.021	0.005	0.287
DHA						
High	1.00	1.00	1.00	1.00	1.00	1.00
Medium	1.18 (0.80–1.89)	1.24 (0.72–2.23)	1.36 (0.46–3.99)	1.28 (0.35–4.37)	1.91 (1.25–2.93)	1.44 (0.82–2.53)
Low	1.32 (0.87–2.03)	1.31 (0.73–2.30)	2.71 (1.01–7.08)	2.58 (0.77–7.69)	2.43 (1.61–3.67)	1.86 (1.07–3.23)
P-trend^†^	0.184	0.396	0.037	0.111	<0.001	0.026
25(OH)D						
High	1.00	1.00	1.00	1.00	1.00	1.00
Medium	1.40 (0.91–2.17)	1.49 (0.85–2.61)	2.11 (0.70–6.32)	2.42 (0.68–8.59)	1.61 (1.05–2.46)	1.31 (0.75–2.29)
Low	1.66 (1.08–2.54)	1.60 (0.89–2.89)	3.03 (1.07–8.64)	1.94 (0.54–6.95)	2.59 (1.73–3.86)	1.96 (1.10–3.49)
P-trend^†^	0.020	0.118	0.035	0.378	<0.001	0.017

*Adjusted for gender, age, marital status, days spent out on the land, feeling of being alone, income and smoking.

^†^ Test for trend over tertiles.

### 25(OH)D

There were no significant gender differences in 25(OH)D levels within age categories. However, those reporting SPD had significantly lower 25(OH)D values (48.7 nmol/L, SD: 28.2) than those not reporting SPD (60.2 nmol/L, SD: 34.3; p < 0.01). Mean levels of 25(OH)D by first, second and third tertile were 25.9 nmol/L, SD: 7.7, 52.6 nmol/L, SD: 8.3 and 97.5 nmol/L, SD: 25.6, respectively. There were significant associations between low 25(OH)D levels and SPD in the unadjusted and adjusted logistic regression analysis. We also found significant associations in the age-stratified analyses and significant trends over tertiles (Table 4). Finally, we observed significant trends over biomarker tertiles for age, marital status, days spent on the land, smoking and K6 score (Table 3).

## Discussion

In this cross-sectional survey of Inuit in northern Canada, we found a relationship between low n-3 FA and 25(OH)D values and increased OR for serious psychological distress. SPD was associated with being female, younger age, not being married or living with a common-law partner, spending fewer days out on the land, feelings of being alone, smoking and low income. Further, n-3 FAs and 25(OH)D were inversely related to SPD, especially among older Inuit. Of course, cultural practices are also important for mental health and it may be that our biomarkers serve as proxies for cultural activities related to food collection, sharing and consumption that increase both biomarker levels and psychological well-being [26].

### N-3 fatty acids

We found significant associations between low levels of n-3 FAs and risk of SPD. However, it appeared that older participants accounted for the association. In the age ≥ 50 years category, low and medium levels of various n-3 FAs were almost all significantly associated with higher ORs for SPD, while this was not the case for those under 50 years of age. It is possible that n-3 FAs co-varies with other factors not captured by our measures that might also explain the lower rates of SPD among older participants. N-3 LC-PUFAs (DHA, EPA and DPA n-3) showed the strongest association with SPD. However, analyses stratified on age found nothing significant in the age < 50 years category. On the other hand, low levels of n-3 LC-PUFAs in the age ≥ 50 years category more than doubled the OR for SPD compared with only EPA and DHA in combination. An unexpected finding was that the addition of DPA n-3 to EPA and DHA made the association with SPD stronger. DPA n-3 is an elongated metabolite of EPA that has not received as much study as EPA and DHA because it is not readily available in purified form [28]. Of note, DPA n-3 content in seal is much higher than in common fish species, and seal meat has been a central component of traditional Inuit diet [29]. Thus, the increased strength of the association between n-3 LC-PUFAs and SPD, compared with EPA+DHA alone, could be explained by the additional DPA n-3 gained by seal consumption. DPA n-3 may influence underlying neurobiological processes, which reduce the risk for SPD. Alternatively, DPA n-3 level may be a reflection of the dietary practices of older Inuit who consume more seal meat and other traditional foods. Older Inuit DPA n-3 values were significantly higher compared with younger Inuit (p-value <0.001). However, patterns of food consumption are part of broader cultural practices. Older Inuit also receive traditional food as a sign of respect. Hence, DPA n-3 levels could be a proxy for social connection and support, which in itself could reduce SPD prevalence.

DHA also showed a significant trend over tertiles in the multivariable analysis and it was the only FA that showed a significant increased OR for SPD in the overall sample. DHA is the predominant PUFA in the brain and found in especially high concentrations in neuron-dense grey matter [30]. However, the role of levels of DHA in relation to neuropsychological functioning and mental health is far from established [31].

### 25(OH)D

Sufficient vitamin D levels have been suggested as 25(OH)D levels exceeding 50 nmol/L [32]. The mean 25(OH)D level in the first tertile was below recommendations at 25.9 nmol/L, well into the deficiency range, while mean level in the second and third tertiles exceeded recommendations. In our sample, 45.9% had 25(OH)D levels below the 50 nmol/L threshold. Among Inuit aged ≥ 50 years only 11.5% were of low status and 88.5% had values above 50 nmol/L, while only 40.3% had levels > 50 nmol/L and 60.7% were low in the age < 50 category. In Edmonton at 52°N, sun exposure is insufficient for the production of previtamin D_3_ from October through March [33]. All our participants reside at latitudes above 54°10´N with even less sunlight to rely on for vitamin D production. Further, nutritional dense traditional food consumption is declining [2], which may render Inuit at risk for developing vitamin D deficiency associated diseases. We observed significantly increased ORs for SPD in the low level 25(OH)D categories in both unadjusted and adjusted analysis as well as a significant trend over tertiles. We observed the same tendency in the age-stratified models, but found significant results only in the bivariate models. This may be due to the age-related 25(OH)D level differences such that the variation within age cut-offs is not large enough for statistical assessment.

### Culture and mental health

Inuit have experienced tremendous changes as colonialism, sedentarization, and westernisation have influenced their way of life with emphasis on individualism, wage-based economy and consequent cultural discontinuity and social stressors [34]. The Inuit concept of the person has been described as *ecocentric* [7], and It is commonly held that being out on the land promotes well-being and can prevent sickness. Consumption of traditional country foods is thought to promote health, strength and vitality. Food sharing with others also plays an important role in relationships, and it acts as an identity marker. We found a strong association between time spent on the land and SPD but this disappeared when other variables were controlled. Those who benefit from being out on the land may have been under-represented in our sample because they were out on the land at the time of the survey.

In an earlier survey of Inuit in Nunavik, participation in hunting was associated with lower risk of suicide attempts among female youth [20]. More females than males participated in the IPY survey. A higher female participation ratio is common in survey-based studies and our findings are no exception. In many populations, women may be more likely than men to report psychological distress. However, risk of death by suicide is higher among males, which does not correspond to the relatively higher SPD rate among women [35]. It is possible that men are reluctant to disclose their distress and that the actual prevalence of SPD is higher in men than that observed [36]. There also may be aspects of Inuit culture not captured by our measures that influence the risk of SPD and that are also associated with the biomarkers we assayed. For example, is possible that reduced psychological distress as well as high biomarker levels may both be consequences of adherence to tradition, traditional food consumption and Inuit culture, which enhance social cohesion and support through community feasts and sharing of traditional foods.

### Strengths and limitations

Our findings are in accord with research conducted in Nunavik, another Canadian Inuit region [26]. Survey participant’s natural high biomarker levels attained by a high traditional, marine food intake provided an opportunity to investigate associations between psychological distress and biomarker levels that exceeded levels commonly found in the literature [37]. Among the strengths of the present study are the use of a large, representative community sample. One limitation of the survey data is that they were gathered during the relatively sunny, warm months of August through October. This time of sampling may hide a higher SPD prevalence during the darker, colder time of the year associated with Seasonal affective disorder (SAD), defined as depression with seasonal pattern most often expressed in autumn and winter months, which is known to be more prevalent at higher latitudes [38]. Hence, a higher SPD prevalence might have been identified had the survey been conducted during the darker winter months, a period when diet also shifts reflecting change patterns of hunting and harvesting.

In the IPY survey, the questions on psychological distress were administered via a separate confidential questionnaire. Given that only 65.5% of the total sample answered all the K6 questions, the actual SPD prevalence may be higher due to participants' reluctance to disclose personal and sensitive information about their mental health. This reluctance might apply especially to older men, who reported low levels of SPD.

Finally, the cross-sectional nature of the data does not allow any causal inference about the relationship between nutritional biomarkers and mental health, and the lack of variability in the younger age group makes the statistical interpretation challenging. Age and biomarker levels are closely linked, which makes it difficult to discern any specific effects of cultural transitions on biomarkers.

## Conclusion

We found a general trend towards higher risk for serious psychological distress with decreased n-3 fatty acid and 25(OH)D levels. The associations were stronger among participants aged ≥ 50 years. These observations may have both cultural and biological explanations. Our results are consistent with current initiatives in many Inuit communities to make traditional subsistence-related cultural values, practices and activities present in everyday life as part of a holistic approach to promote better mental health.
